# Effect of Hydrogen Bonding on Dynamic Rheological Behavior of PVA Aqueous Solution

**DOI:** 10.3390/gels8080518

**Published:** 2022-08-19

**Authors:** Qingsheng Ni, Weijuan Ye, Miao Du, Guorong Shan, Yihu Song, Qiang Zheng

**Affiliations:** 1 State Key Laboratory of Chemical Engineering, College of Chemical and Biological Engineering, Zhejiang University, Hangzhou 310027, China; 2 MOE Key Laboratory of Macromolecular Synthesis and Functionalization, Department of Polymer Science and Engineering, Zhejiang University, Hangzhou 310027, China; 3 Institute of Zhejiang University-Quzhou, Quzhou 324000, China

**Keywords:** dynamic rheological behavior, PVA aqueous solution, hydrogen bond, relaxation spectrum

## Abstract

The rheological behavior of polyvinyl alcohol (PVA) aqueous solution is crucial to optimizing the processing technology and performance of PVA products. In this paper, the dynamic rheological behavior of PVA aqueous solution was investigated in detail. PVA solution with a concentration of 10 wt% showed unnormal rheological behaviors, that is, the liquid-like behavior in the high frequency (*ω*) region and the solid-like behavior in the low *ω* region. A storage modulus (*G*′) plateau appears in the relatively low *ω* region as a gel with a network structure. Different from conventional hydrogel, this plateau has a low modulus, and the corresponding size of the relaxation unit is estimated to be 554 nm, being higher than the size of a whole PVA chain. It is believed that the network mesh is formed by the intermolecular hydrogen bonding interactions among PVA chains. The relaxation time of these meshes is longer than the reptation time of a PVA chain. Based on the relaxation spectrum and calculation analysis, it is found that the destruction of intermolecular hydrogen bonds, such as by heating up, adding sodium dodecyl sulfate, and shear operation, will make the relaxation unit (mesh) larger and lead to the left shift of the intersection of *G*′ and loss modulus (*G*″). In a PVA solution with a high concentration, multiple meshes of various sizes could be formed and thus generate multiple relaxation peaks. The large-sized meshes mainly contribute to the left shift of the intersection of *G*′ and *G*″, and the small-sized meshes contribute to the high plateau modulus. The results in this paper offer a new angle to analyze polymer solutions with strong intermolecular interaction.

## 1. Introduction

All rheological behaviors of polymers, whether time-dependent or shear-rate-dependent, arise from the changes in the microstructure of the polymer system. Dynamic rheology, where the variation of the microstructural network is substantially correlated with the macro rheological behaviors of the polymer system, can make us better understand the effect of the original structure on properties. Compared with the steady-shear rheological, the dynamic rheological test is usually measured under the condition of small strain, and the process will not affect or damage the structure of the material itself. This kind of linear/nonlinear viscoelasticity of polymer materials is sensitive to the change in morphology and structure [1,2,3,4].

Conventional polymer solution (semidilute or concentrated) usually shows the dominance of viscosity, i.e., loss modulus (*G*″) beyond the elastic modulus (*G*′) at the low frequency (*ω*) region [5,6]. It can be described by a single Maxwell model, i.e., the slopes of log $G^{\prime }$ and log $G^{\prime \prime }$ versus log ω in the low ω region are close to two and one, respectively [7]. However, the existence of strong intermolecular interactions makes it complex [8,9]. Wu [10,11] investigated the rheological behavior of sodium carboxymethyl cellulose aqueous solution doped with dodecyl trimethyl ammonium bromide (C_12_TAB) and found that the network structure formed by the addition of C_12_TAB becomes more and more compact, the deviation of *G*′ and *G*″ in the low *ω* region from the classic viscoelastic theory increases relevantly. When the concentration of C_12_TAB reaches 10 mmol, the *G*′ of the system is higher than *G*″ over the low *ω* region. Ioannis [12] explored the rheological properties of polyelectrolyte-surfactant mixtures. It was found that with the increase in surfactant concentration, the effect of cross-linking and association caused by surfactant molecules were enhanced, leading to the transition from the dominance of *G*″ to the dominance of *G*′ in the low *ω* region. Li [13] studied the rheological properties of cationic guar gum aqueous solution and discussed the meaning of the intersection point of *G*′ and *G*″. Within the range of the tested system, *G*″ > *G*′ prevails, and viscous always dominates. Ilyin [14] studied the solution of sulfonated polyoxadiazole and found that with the increase in the concentration, the solution changed from *G*″ > *G*′ to *G*″ < *G*′. Kulichikhin [15] studied the rheological properties of a series of acrylonitrile-based copolymers dissolved in dimethyl sulfoxide (DMSO) solutions and found that when the water content in DMSO is up to 7 wt%, *G*′ > *G*″ in low *ω* region appeared.

Polyvinyl alcohol (PVA) is recognized as one of the vinyl polymers resoluble in water and degradable in the presence of suitably acclimated microorganisms [16]. In most cases, PVA is processed and used in a state of an aqueous solution. Thus, the rheological behavior of the PVA aqueous solution is crucial to optimizing the processing technology and performance of PVA products [17]. It has been found that the dynamic rheological behavior of PVA aqueous solution showed $G^{\prime }$ > $G^{\prime \prime }$ and frequency independence prevailed in the low *ω* region as a weak gel, but the in-depth systematic discussion is insufficient [18,19,20]. Existing reports showed that the rheological behavior of PVA solution is greatly influenced by the strength of the hydrogen bonding between the polymer chains and water molecules [18,21]. The intermolecular hydrogen bonds in PVA solution can form a physically cross-linked network [22], and so they make the PVA chains relax slowly at low frequency. In addition, the relaxation spectrum is the most general method to describe the dependence of material viscoelasticity on time or frequency. Through the study of the relaxation spectrum, the distribution of relaxation time (*τ*) and the contribution of various motion modes to macroscopic viscoelasticity can be fully grasped, so as to provide an effective way for studying the microstructure of viscoelastic materials [7,23,24].

In this paper, the dynamic rheological behaviors of PVA aqueous solution under various conditions were investigated in detail. Furthermore, the mesh calculation and relaxation spectrum are used to analyze the effect of pre-shear, temperature, concentration, and additives on the dynamic rheological behavior of PVA solution.

## 2. Results and Discussion

### 2.1. Dynamic Rheological Behavior of PVA Aqueous Solution

**Figure 1:**
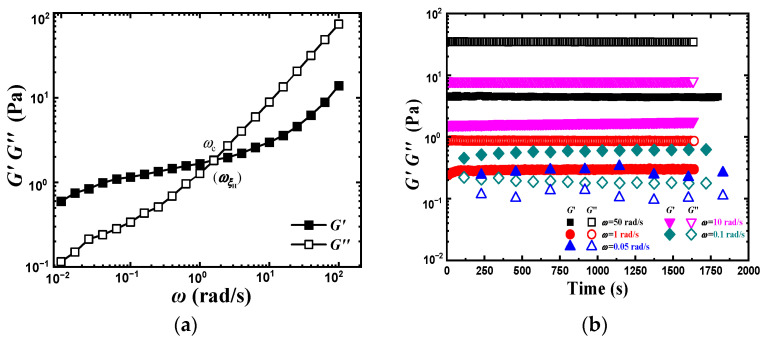
Dynamic rheological behavior of 10 wt% PVA aqueous solution. (**a**) Frequency scanning result. (**b**) Timescanning result.

The critical entangled concentration ($C_{e}$) of PVA aqueous solution is very low due to the intermolecular hydrogen bond. According to Ref. [22], $C_{e}$ can be calculated from,

(1) $$C_{e}=\rho M_{e}/M_{w}$$

in which, *M_e_* (3.75 kg/mol) and *M_w_* (108 kg/mol) are the entangled molecular weight and weight-average molecular weight of PVA, and *ρ* is the density of PVA, i.e., 1.27 g/mL [25]. Then, *C_e_* = 4.4 wt% is obtained. In this paper, the dynamic rheological behavior of a semidilute entangled solution, i.e., PVA aqueous solution with a concentration of 10 wt%, was investigated in a meticulous and deep-going way. Figure 1a shows the dynamic frequency scanning curve of 10 wt% PVA aqueous solution at 298 K. There is an intersection (*ω*_c_) point between *G*′ ~ *ω* and *G*″ ~ *ω* curves. Different from conventional polymer solution, *G*′ > *G*″ at *ω < ω*_c_, while *G*′ < *G*″ at *ω* > *ω*_c_. A *G*′ plateau with low modulus appears in the relatively low *ω* region as a gel. The frequency corresponding to the intersection point of *G*′ and *G*″, $\omega_{c}$, is 1.6 rad/s. Namely, $1/\omega_{c}=0.6\;s$, which is much longer than the reptation time ($\tau_{\mathrm{rep}}$) of a whole chain in entangled concentrated polymer solution (usually 10^−2^ s) [26]. To further confirm this phenomenon, the dynamic time scan tests of 10 wt% PVA aqueous solution under different *ω* were executed, as shown in Figure 1b. Both *G*′ and *G*″ maintain unchanged within 1800 s. Interestingly, *G*′ > *G*″ prevails also at low *ω* (0.1 and 0.5 rad/s), while *G*′ < *G*″ at high *ω* (10 and 50 rad/s), which is consistent with Figure 1a. As we know, there are strong intra- and intermolecular hydrogen-bonding within PVA aqueous solution that may lead to complex rheological behavior. Especially, intermolecular hydrogen-bonding favors to form network structure.

**Figure 2:**
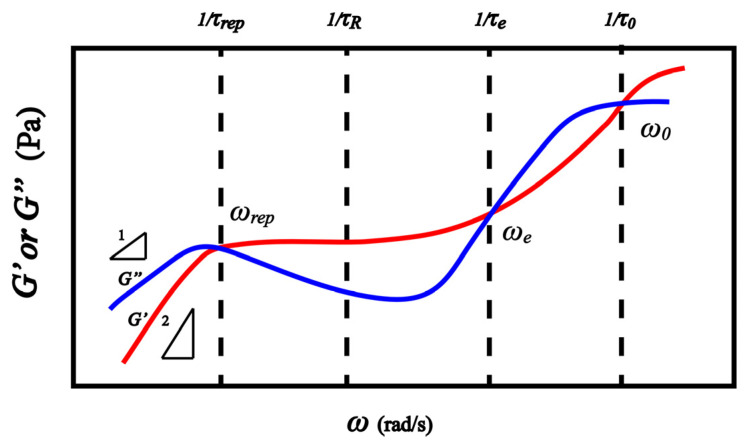
Sketch of dynamic frequency sweep curve of polymer chain in a wide frequency range at room temperature.

In order to understand the unnormal dynamic rheological behavior of PVA solutions, it is necessary to discuss the relaxation behavior of conventional polymers. Generally speaking, the dynamic relaxation behaviors of polymer chains present four regions, as shown in Figure 2. There are three intersection points of *G*′ and *G*″ curves. The first intersection point $\omega_{\mathrm{rep}}$ corresponds to the reptation time of a whole chain $\tau_{\mathrm{rep}}=1/\omega_{\mathrm{rep}}$, the second intersection point $\omega_{e}$ is the reciprocal of the Rouse time ($\tau_{e}$) of an entanglement strand containing *N_e_* monomers, and the third intersection point $\omega_{0}$ relates to the relaxation time $\tau_{0}$ of the Kuhn unit [27,28].

In order to determine the characteristic relaxation of PVA in an aqueous medium, the $\tau_{\mathrm{rep}}$ of the PVA chain in a 10 wt% PVA aqueous solution could be estimated by the following formula [27],

(2) $$\tau_{\mathrm{rep}}=\tau_{0}{\left(\frac{\xi }{b}\right)}^{3}{\left(\frac{N_{e}}{g}\right)}^{2}{\left(\frac{N}{N_{e}}\right)}^{3}$$

*N* is the degree of polymerization of PVA molecular chains, *b* is the size of Kuhn unit of about 1 nm for PVA chains [27], *ξ* is the size of each correlation blob which can be obtained by,

(3) $$\xi \approx b\phi^{-0.76}$$

*g* is the number of monomers in a blob and $N_{e}$ is the number of monomers between entanglements of molecular chains, the order of $N_{e}$ is usually 10^2^ [29]. $\tau_{0}$ is the relaxation time of the Kuhn unit and can be obtained by [27]

(4) $$\tau_{0}=\frac{\eta_{s}b^{3}}{kT}$$

where *k* is the Boltzmann constant, *T* is the Kelvin temperature, $\eta_{s}$ is the viscosity of the solvent, the water at 298 K is about 0.893 × 10^−3^ Pa·s, and $\tau_{0}$ can be obtained as 7.33 × 10^−10^ s. Combined with the above parameters, $\tau_{\mathrm{rep}}$ can be estimated as 0.04 s ($\omega_{\mathrm{rep}}$ = 25 rad/s) at *N*_e_ = 50 and 0.0041 s ($\omega_{\mathrm{rep}}$ = 245 rad/s) at *N*_e_ = 500. Note that these estimated $\tau_{\mathrm{rep}}$ are actually much smaller than the 1/$\omega_{c}$ (=0.6 s) of Figure 1, implying that the size of the relaxation unit near $\omega_{c}$ is greater than that of the whole chain. It is speculated that the relaxation unit near $\omega_{c}$ is attributed to the network structure formed by the intermolecular hydrogen bonding. The network mesh may be composed of several chains, and its size ($\xi_{H}$) may be larger than one PVA chain. Thus let $\omega_{c}=\omega_{\xi_{H}}$, and its characteristic relaxation time is named as $\tau_{\xi_{H}}$($\tau_{\xi_{H}}=1/\omega_{\xi_{H}}$). That is $\omega_{\xi_{H}}<\omega_{\mathrm{rep}}$ and $\tau_{\xi_{H}}>\tau_{\mathrm{rep}}$.

Next, we take the above results as the basis to calculate the plateau modulus at low frequency. Comparing the schematic in Figure 2 with the dynamic rheological behavior of the PVA aqueous solution shown in Figure 1, it can be found that the $\omega_{\xi_{H}}$ is similar to the $\omega_{e}$ of Figure 2, where *G*′ > *G*″ on the left of $\omega_{e}$ and *G*′ < *G*″ on the right of the $\omega_{e}$. As for general entangled polymer solutions, the plateau modulus (G(ϕ)) caused by molecular entanglement can be obtained by: [27]

(5) $$G(\phi )\approx \frac{kT\phi }{\xi^{3}N_{e}(\phi )}\approx \frac{kT\phi }{\xi^{3}N_{e}}\phi^{3v/3v-1}$$

where *ϕ* is the polymer volume fraction, *ν* is the exponent related to the solvent, which is 0.588 for good solvent and 0.5 for θ-solvent. Different from the plateau modulus of general polymer solution and melt, there is a strong intermolecular hydrogen bond within PVA chains to form a network structure. We believe that the intersection point ($\omega_{\xi_{H}}$) presented in Figure 1a actually reflects the relaxation behavior of the network mesh composed of several chains, which are connected by the intermolecular hydrogen bond. Thus, the plateau modulus cannot be obtained directly from the above equation but can be obtained by the transformation of Equation (5) where $\xi^{3}$ is replaced by the volume of a whole chain *D*_h_^3^ because of $\omega_{\xi_{H}}$ < $\omega_{\mathrm{rep}}$, where *D*_h_ is the hydrodynamic diameter of PVA chains in water and is about 25 nm obtained by dynamic light scattering (DLS) [30].

**Figure 3:**
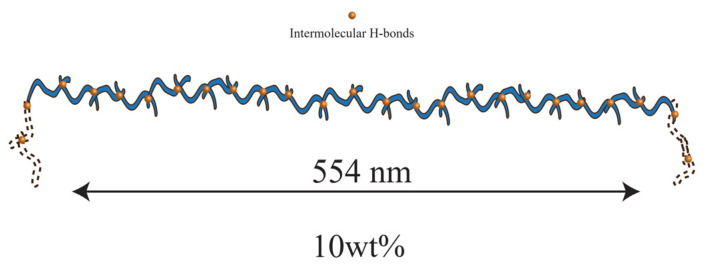
Schematic diagram of hydrogen bond mesh in PVA solution.

Assuming that the size of the intermolecular hydrogen bond network mesh is $\xi_{H}$, we can replace $N_{e}$ with $\xi_{H}/D_{h}$, which is a bit rough but makes the problem concise, and then the relationship between G(ϕ) caused by an intermolecular hydrogen bond and the mesh size $\xi_{H}$ can be obtained as follows,

(6) $$G(\phi )\approx \frac{kT}{D_{h}{}^{2}\xi_{H}}\phi^{1.35}$$

then the $\xi_{H}$ of 10 wt% PVA solution in Figure 1a is obtained as 554 nm. This means that the network mesh formed by the intermolecular hydrogen bond (denoted as intermolecular H-bond mesh) is composed of about 22 PVA chains, where the PVA chains usually present a helical structure in an aqueous solution due to the intramolecular hydrogen bond [31,32]. For convenience, this paper simplifies the spiral chain as depicted in Figure 3. Just like the physical meaning of melt plateau modulus, the plateau modulus of $\omega_{\xi_{H}}$ can be roughly considered as the *kT* order of the number of intermolecular hydrogen bond entanglements per unit volume.

### 2.2. Weak Intermolecular Interactions—PEG Solution

**Figure 4:**
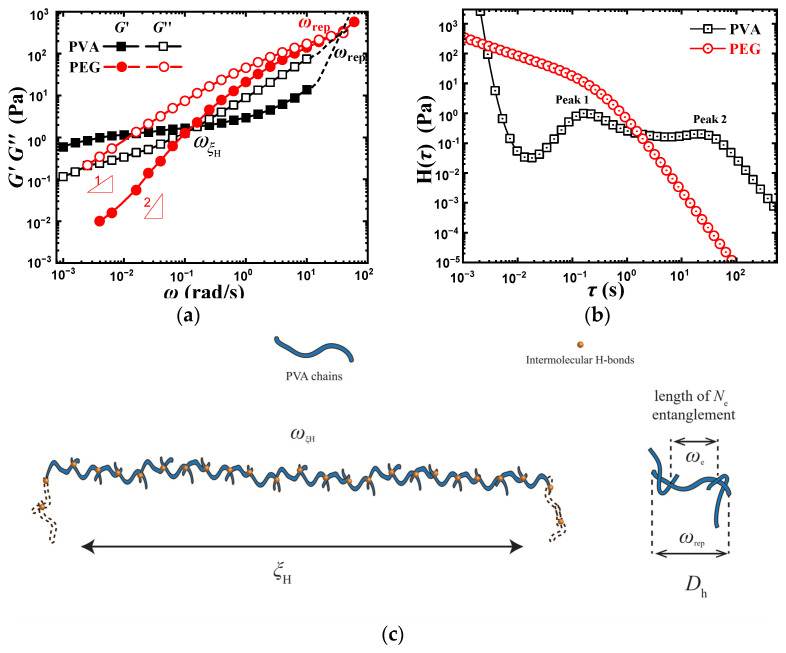
Dynamic test result and the corresponding relaxation spectrum of 10 wt% PVA and 10 wt% PEG solution. (**a**) Dynamic rheological behavior. (**b**) Relaxation spectrum. (**c**) Schematic diagram of relaxation units at $\omega_{\xi_{H}}$, $\omega_{\mathrm{rep}}$ and $\omega_{e}$.

Since the unusually dynamic rheological behavior of PVA solution is caused by the strong intermolecular hydrogen bond, we choose polyethylene glycol (PEG) without hydroxyl group but a similar structure to PVA as a comparison. The molecular weight of both is 70,000 g/mol. PVA solution can form intramolecular and intermolecular hydrogen bonds due to the presence of hydroxyl groups, while PEG chains can only form hydrogen bonds with water [33]. The dynamic rheological behaviors of the two solutions are shown in Figure 4. The slope of *G*′~*ω* and *G*″~*ω* of PEG solution is close to one and two respectively in the low *ω* region, reflecting the general characteristics of polymer solution, while that of PVA solution is much smaller and the platform appears, reflecting the characteristics of network structure [34]. Moreover, the *G*″ of PEG solution is higher than that of PVA solution in measuring the *ω* region despite their similar *M*_w_.

In general, the mechanical energy imparted to the sample either is stored elastically, which yields the storage modulus *G*′, or is dissipated as heat through the motion of molecules, which is the loss modulus *G*″ [3], and the dissipated energy of *G*″ in high *ω* is actually dependent on polymer substrates which can be interpreted by loss area (LA) theory. LA theory considered that each part of the molecular chain contributes a specific value to *G*″ through different vibrate ways of molecules [35,36,37], which can be expressed by the following formula,

(7) $$LA=\sum_{i=1}^{n}\frac{LA_{i}\cdot M_{i}}{M}=\sum_{i=1}^{n}\frac{G_{i}}{M}$$

where *M_i_* is the molecular weight of the *i*th group in the repeating unit, *M* is the molecular weight, *G_i_* is the molar loss constant for the *i*th group, *LA_i_* is the loss area contributed by the *i*th group, and *n* represents the number of moieties in the monomer. According to the reference [35], the *G_i_* of the PEG monomer is 397.8 (GPa·K) (g/mol) while the *G_i_* of the PVA monomer is 171.8 (GPa·K) (g/mol), which resulted in the larger *G*″ of PEG solution than that of PVA solution. The intersection point presented in the PEG solution at about 200 rad/s actually reflects the relaxation time of the entire PEG molecular chain, i.e., $\tau_{\mathrm{rep}}$ [26,27]. It implies that the $\tau_{\mathrm{rep}}$($\omega_{\mathrm{rep}}$) of the PVA molecular chain may also be located in the intersection of the dashed line, as shown in Figure 4a. It reveals that the size of the relaxation unit at $\omega_{\xi_{H}}$ in the PVA solution may be larger than the entire PVA chain, which is strong evidence to support the calculated result discussed in the previous section. The schematic diagrams of relaxation units corresponding to $\omega_{\xi_{H}}$, $\omega_{\mathrm{rep}}$ and $\omega_{e}$ are shown in Figure 4c.

According to Figure 4a, the relaxation spectrum (H(*τ*)~*τ*) is obtained and shown in Figure 4b. For the PEG solution, the relaxation spectrum shows a plateau in the small *τ* region, which reflects the reptation relaxation of PEG chains. For the PVA solution, there are two small peaks in the region of 10^−1^~10^2^ s, where a plateau appears between the two peaks. This is considered the relaxation of intermolecular H-bond mesh, and the corresponding relaxation time $\tau_{\xi_{H}}$ is larger than that of the whole chain, $\tau_{\mathrm{rep}}$. Since the PVA sample is polydispersed, the calculation of $\omega_{\xi_{H}}$ based on the dynamic test actually reflects the average value of the intermolecular H-bond meshes in the PVA solution. The formation of hydrogen bonds can be considered as a random process to make the mesh size present a wide distribution, and the wide plateau represents the relaxation with different sizes of the meshes.

### 2.3. Pre-Shear

**Figure 5:**
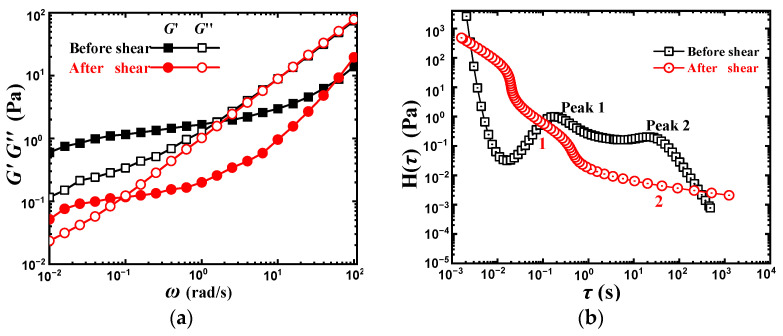
Dynamic rheological behavior and the corresponding relaxation spectrum of 10 wt% PVA before and after shear. (**a**) Dynamic rheological behavior. (**b**) Relaxation spectrum.

In order to better explore the effect of intermolecular hydrogen bonds on the dynamic rheological behavior of PVA solution, a pre-shear experiment was carried out. A dynamic frequency scanning operation was operated firstly, then a steady shear procedure under 0.1 s^−1^ for 10 min was applied to destroy the intermolecular hydrogen bond, and finally, the next dynamic frequency scanning operation was immediately conducted, and the results were shown in Figure 5a.

Both *G*′ and *G*″ are decreased after shearing at the lower *ω* region, reflecting the destruction of the intermolecular hydrogen bond network. While *G*″ curves almost overlap in the *ω* > 2 rad/s region, which means that the destruction of the intermolecular hydrogen bond will not influence the relaxation at high *ω*. In other words, the intermolecular hydrogen bond network structure in PVA solution mainly increases the *G*″ in the low *ω* or long time region. This increase in *G*″ might be summarized into two reasons. The first is that the connections between molecular chains are reinforced by intermolecular hydrogen bonds, thus increasing the frictional heat generated by the motions of molecular chains. The second is that the formation and destruction of hydrogen bonds are in dynamic equilibrium within a certain *ω* scope, and the applied energy will be dissipated in the hydrogen bond destruction, thus leading to the appreciable increase in *G*″ [38]. Different from *G*″, *G*′ has a significant decrease in the most measured *ω* range, indicating that *G*′ is more sensitive to microstructure change than *G*″. The destruction of the intermolecular hydrogen bond caused the sharp decrease in *G*′ value and shortened *G*′ plateau, suggesting that the abnormal *G*′ > *G*″ at the low *ω* range in Figure 1a arose from the intermolecular hydrogen bond among PVA chains. In addition, the plateau modulus actually reflects the number of entanglements in the system if a polymer chain is completely flexible. The intermolecular hydrogen bonds between PVA chains increased the number of entanglement points and resulted in the increase in *G*′ [29].

The mesh size $\xi_{H}$ of the unsheared system is calculated as 554 nm, while it is 8430 nm for the after-sheared system, which implies that the pre-shear operation really destroyed the original denser intermolecular hydrogen bond network. Figure 5b gives the relaxation spectrum corresponding to Figure 5a, the shear operation makes the original peak one reduced into a relatively narrow plateau one, and peak two becomes a wide but weak plateau two. At the same *τ*, peak one (before shearing) and plateau one (after shearing) exhibit almost the same modulus, which is consistent with the dynamic rheological behavior in the high *ω* region, as shown in Figure 5a. While plateau two has a much smaller modulus than that of peak two. The weakening of peak two into the wide plateau two means the decrease in the intermolecular hydrogen bond structure density, leading to a transformation of the dense network ($\xi_{H}$ = 554 nm) into a loose network structure with a larger mesh size ($\xi_{H}$ = 8430 nm).

### 2.4. Temperature

**Figure 6:**
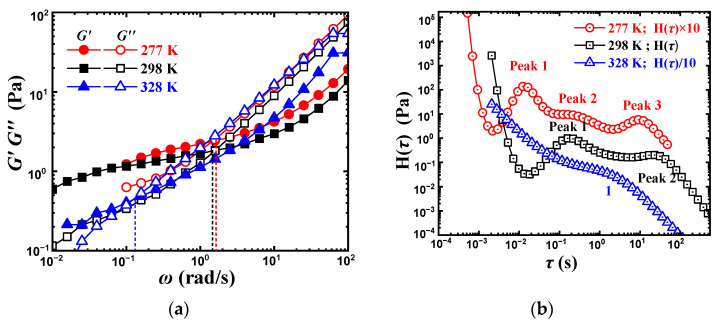
Dynamic rheological behavior and the corresponding relaxation spectrum of 10 wt% PVA at different temperature. (**a**) Dynamic rheological behavior. (**b**) Relaxation spectrum.

Since the hydrogen bond is very sensitive to temperature, the dynamic rheological behavior of PVA solution under different temperatures was carried out, as shown in Figure 6a. Both *G*′ and *G*″ in the low *ω* region decreased with the increase in temperature, reflecting the destruction of the intermolecular hydrogen bond network structure. Furthermore, *G*′ changes remarkably, while *G*″ changes relatively less and almost coincide in the high *ω* region. The $\omega_{\xi_{H}}$ moves to the left when the temperature increases from 277 to 328 K. The $\xi_{H}$ at 298 K is 554 nm, while it is 2200 nm at 328 K and 394 nm at 277 K. The weak intermolecular hydrogen bonds under higher temperatures form a network structure with a larger mesh size that exhibits a larger relaxation time.

Figure 6b gives the corresponding relaxation spectrum from Figure 6a. Let the relaxation spectrum at 298 K as the basis, a wider plateau composed of peak one, peak two, and peak three at 277 K, appears, implying that low temperature can promote the formation of intermolecular hydrogen bonds and maintain the high modulus within a wide relaxation time. While at 328 K, a narrower plateau appears, which is related to the destruction of intermolecular hydrogen bonds. $\omega_{\xi_{H}}$ at 277 K is indeed close to that at 298 K despite of the small-sized mesh structure (394 nm), and the plateau modulus increases slightly, which also reveals that the small-sized intermolecular hydrogen bonding mesh structure contributes more to the platform modulus, while the large-sized hydrogen bonding mesh structure let $\omega_{\xi_{H}}$ shift towards left.

### 2.5. Concentration

**Figure 7:**
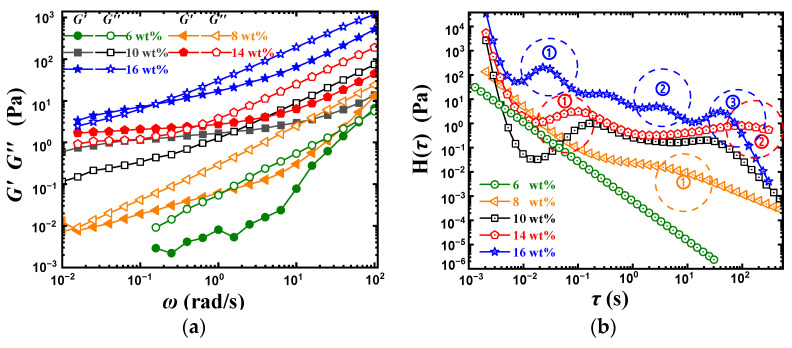
Dynamic rheological behavior and the corresponding relaxation spectrum of PVA solution with concentrations. (**a**) Dynamic rheological behavior. (**b**) Relaxation spectrum.

This paper mainly discusses the dynamic rheological behavior of PVA solution, of which the concentration is the biggest characteristic parameter compared with melts. The effect of PVA concentration on the dynamic rheological behavior is shown in Figure 7a.

With the increase in concentration, both *G*′ and *G*″ increased, especially the *G*″ increased more rapidly. From the contribution of the groups on the polymer chain to the *G*″, it can be seen that the higher concentration corresponds to a larger $\sum G_{i}$, and the larger $\sum G_{i}$ shows that more energy is dissipated in vibrations of monomers and friction between molecules, which results in the rapid increase in the loss modulus *G*″. Moreover, the enhanced effect of intermolecular hydrogen bonding and molecular entanglements can also affect *G*″ in low *ω*, and the enlargement of *G*′ should be attributed to the stronger elastic cross-linking network accompanied by the increase in concentration [39,40,41].

In addition, with the increase in concentration in the range of 6–16 wt%, the intersection point $\omega_{\xi_{H}}$ has a trend of first right shift and then left shift. The parameters of PVA solutions with different concentrations obtained by Equation (6) are listed in Table 1. 

Firstly, the right-shift of $\omega_{\xi_{H}}$ in the concentration range of 6–10 wt% should be discussed. It can be seen in Table 1 that the increase in concentration within the 6–10 wt% $\xi_{H}$ has a significant decline. Similar to the previous discussion, the larger $\xi_{H}$, namely the larger relaxation unit, brings a small density of intermolecular hydrogen-bond networks and results in a decrease in platform modulus [13]. The right shift of $\omega_{\xi_{H}}$ or the decrease in relaxation time in the concentration range of 6–10 wt% is mainly due to the decrease in mesh size. Compared with the plateau composed of two intermolecular hydrogen bond peaks in the relaxation spectrum of 10 wt% PVA solution, the small-sized intermolecular hydrogen bond relaxation peak disappears in 8 wt% PVA solution, only leaving a narrow plateau with low modulus.

For the 10–16 wt% PVA solution, the $\omega_{\xi_{H}}$ gradually shift to the left with the increase in concentration. However, it can be seen from Table 1 that with the increase in concentration, the $\xi_{H}$ slightly decreases in concentration, which suggests a right-shift of $\omega_{\xi_{H}}$. Here, we have to use the relaxation spectrum for further analysis, as shown in Figure 7b. With the increase in PVA concentration, the number of relaxation peaks increases from zero peaks of 6 wt% to four peaks of 16 wt%, and these peaks further connect into a wide relaxation plateau. Multiple relaxation peaks mean multiple relaxation units of different sizes. It is considered that the increasing PVA concentration results in the growth of the number of PVA chains per unit volume. It is favorable to form intermolecular H-bond meshes of different sizes. The peak at the shorter time region reflects the relaxation of smaller-sized intermolecular H-bond meshes, while the peak at longer relaxation time reflects the relaxation of larger-sized intermolecular H-bond meshes. With the increase in PVA concentration, the smaller and larger intermolecular H-bond meshes in the solution are formed at the same time, which the larger meshes will lead to the left shift of $\omega_{\xi_{H}}$, and the smaller meshes will increase the platform modulus and make the calculated $\xi_{H}$ smaller. That is to say, the larger-sized intermolecular H-bond meshes mainly contribute to the increase in relaxation time (low $\omega_{\xi_{H}}$), while the smaller-sized intermolecular hydrogen bonding meshes mainly contribute to the plateau modulus (high intermolecular H-bond mesh density).

### 2.6. Hydrogen Bond Destruction Reagent—SDS

**Figure 8:**
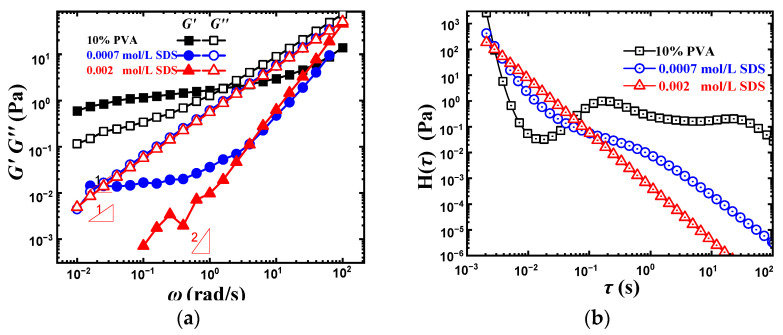
Dynamic rheological behavior and the corresponding relaxation spectrum of 10 wt% PVA with different SDS concentration. (**a**) Dynamic rheological behavior. (**b**) Relaxation spectrum.

It has been reported that sodium dodecyl sulfate (SDS) can destroy the intermolecular and intramolecular hydrogen bond in PVA solution [42]. In this section, SDS was added to further investigate the effect of hydrogen bonds on the dynamic rheological behavior of PVA solution, and the results are shown in Figure 8a.

Critical associated concentration (CAC), i.e., the threshold of concentration for surfactant binding to the polymer chain, is 0.002 mol/L for the PVA-SDS system [42]. In order to weaken the adsorption effect of the SDS and PVA molecular chain itself and better explore the effect of SDS breaking hydrogen bond, the SDS concentration was chosen to be below 0.002 mol/L. With the SDS added, both *G*′ and *G*″ decreased, and the reduction of *G*′ was much more obvious. The slope of log *G*″ versus log ω is close to one when the SDS concentration is 0.0007 and 0.002 mol/L, showing the characteristics of conventional polymer solution, which reflects the destruction of intermolecular hydrogen bonds. The $\xi_{H}$ of 0.0007 mol/L SDS-PVA solution is about 68,800 nm, being much larger than that of pristine PVA solution, which reflects the very weak intermolecular hydrogen bonding interaction in the solution, and the large $\xi_{H}$ gives rise to the left shift of $\omega_{\xi_{H}}$.

Figure 8b gives the corresponding relaxation spectrum. When the SDS concentration was 0.0007 mol/L, the two obvious intermolecular hydrogen bond peaks disappeared, only showing a very weak and narrow plateau. When the SDS concentration increased to 0.002 mol/L, all the peaks disappeared, meaning the disappearance of hydrogen bond networks in the solution, which is consistent with the dynamic rheological behavior in Figure 8a.

## 3. Conclusions

In summary, the unnormal dynamic rheological behavior of PVA aqueous solution primarily comes from the intermolecular hydrogen bonding interaction, which favors the formation of a network structure and generates the *G*′ plateau in the low *ω* region as a weak gel. According to the estimation, the size of the network mesh of 10 wt% PVA is 554 nm, being higher than the size of a whole chain. It suggests that the network mesh formed by the intermolecular hydrogen bonding is composed of several PVA chains. The intersection point of *G*′ and *G*″ in the PVA solution is confirmed as the mean distribution of the relaxation time of these intermolecular H-bond meshes. Shearing operation can destroy the intermolecular hydrogen bonding mesh structure and increase the mesh size. Lowering temperature can enhance the intermolecular hydrogen bonding interaction and decrease the mesh size, while the increasing temperature can destroy the intermolecular hydrogen bonding network and prolong the relaxation time of the mesh. In a high-concentration PVA solution, multiple meshes of various sizes could be formed and thus generate multiple relaxation peaks. The large-sized intermolecular H-bond meshes mainly contribute to the left shift of $\omega_{\xi_{H}}$ and the small-sized meshes contribute to the high plateau modulus.

## 4. Materials and Methods

### 4.1. Materials

The PVA used was purchased from Sinopharm Chemical Reagent Co., Ltd. (Shanghai, China). Its polymerization degree is 1750 ± 50, and the alcoholysis degree is 98~99%. The analytically pure polyethylene glycol (PEG) used was purchased from Shanghai Mokai Biotechnology Co., Ltd. (Shanghai, China). Its average molecular weight is 70,000 g/mol. The chemically pure sodium dodecyl sulfate (SDS) was purchased from Sinopharm Chemical Reagent Co., Ltd. Moreover, deionized water was used in all solutions.

### 4.2. Preparation of Solution

Take 10 wt% PVA/PEG aqueous solution as an example: 5 g of PVA/PEG powder was added into 45 mL water under stirring and heated to 366 K. After complete dissolution, the solution was naturally cooled to room temperature for subsequent testing.

Take 10 wt% PVA-0.002 mol/L SDS aqueous solution as an example: 5 g of PVA powder and 0.029 g SDS were added into 45 mL water under stirring and heated to 366 K. After complete dissolution, the solution was naturally cooled to room temperature for subsequent testing.

The solution concentrations used in this paper are shown in Table 2.

### 4.3. Dynamic Rheological Behavior

A stress-controlled Discovery Hybrid Rheometer (DHR-2, TA Instruments, New Castle, DE, USA) with a 40 mm cone-plate geometry (cone angle of 2°) was used to measure the dynamic rheological behavior of PVA solution. The strain amplitude (*γ*) was set as 2%, which is in the linear viscoelastic region of the sample. The range of angular frequency was 0.01–100 rad/s.

### 4.4. Relaxation Spectrum

The relaxation spectrum was generated by the fitting analysis of the dynamic test results obtained by TA software. In Maxwell model, *G*′ and *G*″ can be expressed as the following discrete form [27].

(8) $$G^{\prime }(\omega )=\sum_{k=1}^{N}H{\left(\tau \right)}_{k}\frac{\omega^{2}\tau_{k}{}^{2}}{1+\omega^{2}\tau_{k}{}^{2}}$$

(9) $$G^{\prime \prime }(\omega )=\sum_{k=1}^{N}H{\left(\tau \right)}_{k}\frac{\omega \tau_{k}}{1+\omega^{2}\tau_{k}{}^{2}}$$

where, ω is the oscillation frequency, $\tau_{k}$ and $H{\left(\tau \right)}_{k}$ represent respectively the relaxation time and relaxation modulus in the Maxwell element of the *k*^th^ group. Then a minimum value problem is constructed, and *N* groups of $\tau_{k}$ and $H{\left(\tau \right)}_{k}$ can be obtained by substituting *G′* and *G"* data obtained in the experiment into calculation, which is the relaxation spectrum [43]. It is worth noting that the parameter $e^{\pi /2}$ is introduced into the calculation of the relaxation time spectrum, making the display range of relaxation spectrum larger than 1/*ω* in dynamic rheological behavior.

## Figures and Tables

**Table 1:** Mesh size of PVA solution with different concentrations.

wt%	ξ_H_ (nm)
6	−
8	76,400
10	554
14	488
16	181

**Table 2:** Concentration of the measured aqueous solution.

Solution	Polymer Concentration wt%
PEG	10
PVA	6, 8, 10, 14, 16
0.0007 mol/L SDS-PVA	10
0.002 mol/L SDS-PVA	10

## Data Availability

Data available on request from the authors.
